# Metric Learning in Histopathological Image Classification: Opening the Black Box

**DOI:** 10.3390/s23136003

**Published:** 2023-06-28

**Authors:** Domenico Amato, Salvatore Calderaro, Giosué Lo Bosco, Riccardo Rizzo, Filippo Vella

**Affiliations:** 1 Department of Mathematics and Computer Science, University of Palermo, 90123 Palermo, Italy; domenico.amato01@unipa.it (D.A.); salvatore.calderaro01@unipa.it (S.C.); giosue.lobosco@unipa.it (G.L.B.); 2 Institute for High-Performance Computing and Networking, National Research Council of Italy, 90146 Palermo, Italy; filippo.vella@icar.cnr.it

**Keywords:** metric learning, triplet networks, embedding, BreakHis, patient level accuracy, breast cancer imaging, WSI, classification interpretability, visualization

## Abstract

The application of machine learning techniques to histopathology images enables advances in the field, providing valuable tools that can speed up and facilitate the diagnosis process. The classification of these images is a relevant aid for physicians who have to process a large number of images in long and repetitive tasks. This work proposes the adoption of metric learning that, beyond the task of classifying images, can provide additional information able to support the decision of the classification system. In particular, triplet networks have been employed to create a representation in the embedding space that gathers together images of the same class while tending to separate images with different labels. The obtained representation shows an evident separation of the classes with the possibility of evaluating the similarity and the dissimilarity among input images according to distance criteria. The model has been tested on the BreakHis dataset, a reference and largely used dataset that collects breast cancer images with eight pathology labels and four magnification levels. Our proposed classification model achieves relevant performance on the patient level, with the advantage of providing interpretable information for the obtained results, which represent a specific feature missed by the all the recent methodologies proposed for the same purpose.

## 1. Introduction

Van der Laak et al. in [1] point out that the digitisation of patient tissue samples, usually called Whole Slide Images (WSI), enabled the development of a set of techniques in the field of biomedical image analysis under the name of computational pathology. In the histopathology field, deep learning algorithms perform similarly to trained pathologists, but only very few of these have reached a clinical implementation.

The resolution of the WSI image can reach 10,000×10,000 pixels and may present high morphological variance and various artifacts [2]. Due to the general complexity of such kinds of images, the analysis of WSI requires a high degree of expertise and can be very time-consuming. In addition, the complexity of this task is further increased by the need to explore the samples at different magnification scales. As a consequence, a complete diagnosis is often obtained through a discussion among specialised physicians that compare the outcomes of different medical analyses (not only images).

Due to their dimensions, WSI images are challenging to process as a whole. For this reason, they are broken into patches or tiles and given as input to the machine learning (ML) algorithms for classification purposes. The attribution of the class to the WSI images is then obtained by combining the labels predicted for the related patches [3]. This task is not simple due to the morphological variance inside the WSI; however, patch analysis remains the most-used technique for WSI processing, and the development of patch processing and classification systems is an active research field.

To measure and compare the contribution of such approaches, some datasets have been proposed in the literature. One of them, considered as a standard, is the BreakHis dataset (BH) [4]. This dataset comprises 7909 histopathological images of eight different kinds of breast cancer collected from 82 patients. For a fair comparison among the proposed approaches, the authors provided a specific split into a 5-fold structure. A deeper description of BH is reported in Section 3. Many deep neural networks (DNN) have been proposed for histopathological image classification [1], due to their state-of-the-art performances in the generic problem of image classification. In many cases, the architecture used to classify BH images is very close to the ones proposed for other classification tasks, such as the case of ImageNet Large Scale Visual Recognition Challenge [5], that are based on convolutional layers [6]. These kinds of networks have a common architecture constituted by two parts: a first one devoted to features extraction and a second combining the extracted features for classification.

Despite the relevant performance that deep neural networks (DNNs) can achieve in the image classification task, their adoption in the medical domain is not straightforward because they are *black-box* methods, making it difficult to understand the logic behind specific decisions.

Today explainability and interpretability are an important part of the discussion as well as research work about deep neural systems and their performances [7]. What we need are systems that make consistent decisions using an explainable mechanism and provide information that users can understand and find meaningful, particularly in the medical domain. An example of an interpretability mechanism giving information that is not fully significant is CAM or Grad-CAM [8], which can highlight an image region crucial for the classification result; however, this mechanism can not explain what, in that region, is truly important or whether it fits with the shared medical knowledge [9].

The paper is structured as follows: The following section reports the related works in the field of classification of the BreakHis dataset, and in the interpretable deep ANN, the Section 3 reports the details on the BreakHis dataset, the proposed architecture and the training method; the obtained results and the discussions are reported in Section 4 and Section 5; finally, in Section 6 some conclusions are drawn.

## 2. Related Works

We already proposed some studies on image classification supporting tools based on metric learning with fuzzy techniques [10] or convolutional deep networks [11,12], or X-ray image classification [13]. The issues related to the classification of histological images are due to the variability of the different image acquisition equipment. For example, authors in [14] indicate stain variability as one of the challenges in classification; stain normalisation was also addressed in [15].

In this paper, we focus on the BreakHis dataset [4], which is widely adopted as a workbench and used in all the papers discussed in this section. The structure of the dataset will be discussed in the Section 3.1, but it is necessary to highlight here some characteristics related to the image classes, the magnification level, and the train/test split. The dataset has a hierarchical organisation with two super-classes (Benign and Malignant) and eight sub-classes (four for each super-class). The images in the dataset have four different magnification levels 40×,100×,200×, and 400×, which are hierarchically related. For example, images at 200× will contain details of images at 100× and 40×.

The availability of images at different magnification levels leads to two possible set-ups: The first is the study of magnification-specific (MS) classification when a classifier is trained and tested for each magnification level and the second is the magnification-independent (MI) classification when the classifier is trained and tested using all the images regardless of their magnification level.

The developers of the BreakHis dataset released a 5-fold train/test split that can be used to facilitate the comparison of the results among different approaches [4]. The proposed split is created at *patient level* so that images of the same patient are never in the training and testing set of the same fold run.

This split is often overridden by many authors for various reasons, but in order to make fair and reliable comparisons, its use is highly recommended, as in the case of this manuscript.

The availability of patient information means that the assessment of the results can be performed considering the images related to a single patient (patient-level assessment or PLA) or the single images (image-level assessment or ILA). In this work, we have decided to adopt only the PLA paradigm since it is undoubtedly the most reliable in terms of closeness to the real case of histopathological image classification. The specific formulas of PLA will be discussed in Section 3.

In the following subsections, we survey most of the methodologies proposed so far for the histopathology image classification problem that use the BH dataset, differentiating them in terms of image magnification, whether specific or independent. Special attention will be devoted to the contributions that follow the folds separation suggested by the BreakHis dataset developers, whose results can be directly compared with the methodology we present in this manuscript.

### 2.1. Magnification Independent Methods

Magnification Independent Binary (MIB) classifiers are trained to separate benign from malignant images using all the available images, regardless of magnification factors. Only a few works fall into this category, and only two use the 5-fold split suggested by Spanhol et al. in [4]. In the following, we will first introduce the two works that use this split, while the latter uses a different split.For each examined work, the PLA value is reported. PLA is an interesting comparison measure since it summarizes the accuracy for a patient and is the most realistic measure of the system performance when a new patient is examined. Other metrics, even if not present in the literature works, are computed for the proposed approach and are shown in Section 4.

Bayramoglu et al. [16] propose two convolutional neural networks to classify breast cancer histopathology images for the MIB case. These models are used to predict the malignancy of the input sample (single-task fashion) or both the malignancy and magnification factor of the input (multi-task fashion). For the single task, a PLA of 82% is obtained.

Another model, proposed by Sun et al. [17], uses an approach similar to the one presented in this paper with both MIB and MSB tasks. Authors adopt a siamese and a triplet network to create a new representation of the images, and they use a different loss function that also considers the samples’ imbalance. The images, without any pre-processing, are fed to the network that implements the classification, and the obtained PLA accuracy is about 88%, which is slightly lower than ours.

The last work, proposed by Gupta et al. [18], adopts selected colour and texture descriptors, baseline classifiers such as SVM, kNN, Decision Trees and Discriminant Analysis, fused together with majority voting. For the model validation, a different train/test split was used. A total of 58 patients (70%) were randomly chosen for the training set and the remaining 24 (30%) for the test set and the process was repeated for five trials. For the magnification-independent classification, the model achieves an average PLA of 87.53%.

### 2.2. Magnification Specific Methods

Several methodologies have been proposed for the case of Magnification Specific Binary classification (MSB). We have decided to filter them on the basis of the adoption of the training-test split proposed in [4] since this choice provides a solid comparison process. As a consequence, all the accuracy values of the methodology reported in the following works use the same training-test split. This is the case for the methodology proposed by Sun et al. [17] described in the previous paragraph, which has also been used for the MSB case. The results show an average PLA ranging from ∼87% (400×) to ∼91% (200×) (see Table 1 for details).

Apart from the provided split, the proposers Spanhol et al. have also investigated two approaches based on patches extraction [19].

The patches for training were obtained by two strategies: using a sliding window with 50% overlap or a random extraction of 1000 patches with no overlap check. These strategies were repeated twice to obtain patches of 32×32 pixels and patches of 64×64 pixels. For the classification task, the authors proposed a variant of the AlexNet architecture. The images of the test set were obtained by using patches extracted with a sliding window and no overlap. The final classification of an image was the result of a combination of classification results, each one computed on a single patch. The best results were obtained with the training of 1000 patches of the largest dimension and using the Max Fusion Rule as a combination paradigm. The patient accuracy values for each magnification level range from 84% (200×) to 90% (200×) for PLA (see Table 1 for details).

Spanhol et al. have proposed another solution [20] exploiting the adoption of specific image features, named DeCAF. They are obtained by extracting the outputs of the top 3 layers of a pre-trained AlexNet-like network and using them as the input of a CNN classifier. The experiments were organised considering a patch-based recognition, using a different number of patches ranging from 1 to 16. The achieved accuracy ranges from 82% (400×) to 86% (200×) for PLA (see Table 1 for details).

The idea of DeCAF features has also been investigated by Benhammou et al. [21] using a pre-trained Inception v3 [22]. To obtain the features, during the fine-tuning step, only the weights of the last fully connected layer (situated before the softmax layer) are retrained, while the other net layers are frozen. A pre-processing step on the images based on the mean-pixel subtraction is used. The achieved accuracy ranges from ∼80% (400×) to 87% (40×) for PLA (see Table 1 for details).

Another couple of studies that use pre-defined features was proposed by Song et al. [23]. The two approaches exploit Fisher Vectors encoding [24] as feature representation. In their first approach [23], the authors used the descriptors together with a linear support vector machine. First, the features of VGG-D network, pre-trained on the ImageNet dataset, were extracted and then represented with Fisher vector encoding. Next, an adaptation layer formed by two locally connected layers was adopted, and an additional classification layer was also added. The descriptors obtained from the adaptation layer were used to train linear-kernel support vector machines. The obtained average PLA is bounded by ∼86% (400×) and ∼90% (40×) (see Table 1). The second approach [25] provides a supervised intra-embedding algorithm that uses a neural network to transform the Fisher Vector encoding into more discriminating feature representations. The input images are re-scaled to multiple sizes, and for each re-scaled image, the VGG-VD ConvNet pre-trained on ImageNet is applied so that the last layer produces a feature vector of size 512. The features collected from all the re-scaled images are pooled together to generate the so-called ConvNet-based Fisher Vectors (CVF) encoding of the image. To perform the classification, a Support Vector Machine is used. The average PLA values are bounded by ∼87% (400×) and ∼90% (40×) (see Table 1 for details).

Sudharshan et al. adopted a Multiple Instance Learning (MIL) framework for convolutional neural networks [26]. MIL is concerned with learning from sets (bags) of objects (instances), where the classification label is assigned to the bag, not to the single instance. For the training phase, 1000 patches of size 64×64, are randomly extracted from each image, while for the testing phase, a grid of non-overlapping patches is extracted, yielding around 100 patches per image. Each patch is represented by a specific 162-long feature vector of Parametric-Free Threshold Adjacency Statistics (PFTAS) features. The work presents an evaluation of twelve different MIL variants, both parametric and non-parametric.

The results are provided for two different settings: one where each patient is considered as a bag, considering multiple images for each of them and the second where each image is a bag composed of patches. The best results are obtained at Patient Level using a non-parametric MIL. They range from ∼87% (200×) to ∼92% (40×) and are listed in Table 1 for details.

The papers reported and discussed so far are the ones that can be directly compared with our proposal since all of them share the same dataset split for computing classifier performances. Other train-test splits at the patient level are available, such as the one proposed by Kumar and Rao [27], but it is not actually considered a benchmark dataset. Interested readers could decide to adopt it in their benchmark studies.

As said before, it is very important that the train and test sets must not contain images from the same patients; otherwise, the samples from the same patient could occur in both train and test sets at different magnification levels. This point makes the accuracy artificially higher than the other approaches. Several methodologies overlooking this issue have been proposed. An example is the work in Wei et al. [28], which proposes a CNN architecture trained from scratch with ImageNet first and fine-tuned with the BH dataset, or in Bardou et al. [29], which compared the use of two sets of features, handcrafted and automatically extracted. Other works use GoogleNet [30], different versions of VGG networks [31] and Restricted Boltzmann Machine [32]. Finally, some contributions restrict the study to images at specific magnification sizes, such as [33,34], which used only 40× images.

## 3. Materials and Methods

### 3.1. The BreakHis Dataset

**Figure 1:**
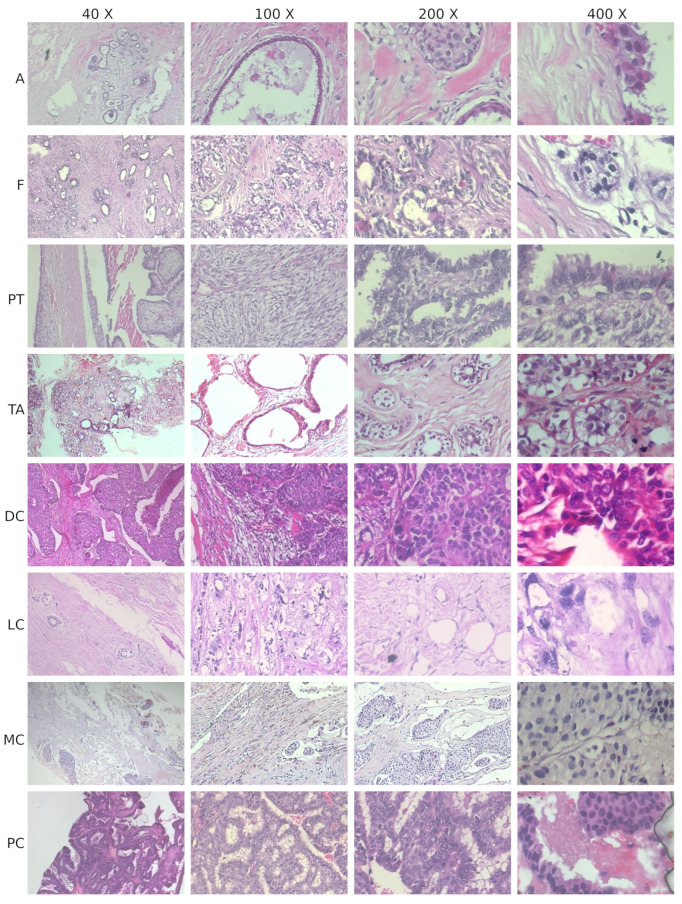
Some images from BreakHis dataset. There is a column for each magnification factor and a row for each subclass: A (Adenosis), F (Fibroadenoma), TA (Tubular Adenoma) and PT (Phillodes Tumor) are benign, DC (Ductal Carcinoma), LC (Lobular Carcinoma), MC (Mucinous Carcinoma) and PC (Papillary Carcinoma) are malignant.

The BreakHis dataset comprises 7909 images at a resolution of 700×460 pixels obtained from tissue samples of 82 breast cancer patients. These samples are divided into two classes, Benign and Malignant, and each of them is separated into four sub-classes, according to the structure in Table 2. The images were acquired at different magnification levels (40×,100×,200×,400×); Figure 1 reports sample images from the dataset.

In this paper, we are only interested in the dichotomy of Benign vs. Malignant, and Table 2 shows that the number of images in the malignant class is double that of the number of benign images.

Although many authors used pre-processing techniques on images, such as stain-normalisation or whitening, we do not consider any of these techniques because our preliminary experiments show that they provide no performance increase. Moreover, these pre-processing techniques require an additional processing time, sometimes very long, and add a new set of parameters: for example, in stain normalisation a reference image must be selected from the training dataset; or in a whitening procedure, color mean and variance must be calculated on available training images.

As said before, the original paper presenting the dataset [4] also proposes a train/test split for the training of a classifier. This split is based on a 70–30% proportion that also considers the patient set to avoid the presence of images of the same patient both in the training and test set.

The availability of this specific partitioning is of great advantage for the assessment of the overall quality and potential limitations of the results, which, in the general case, must be carefully performed by the adoption of specific statistical tools [35].

Using this method, five training/test splits were generated and can be downloaded with the dataset allowing a complete comparison of the classification results.

The classification results will be reported using the so-called PLA [4], i.e., a performance index that takes into account the results on a patient level in the following way:

(1) $$Patient\;Score=\frac{N_{rec}}{N_{P}}$$

where $N_{P}$ is the number of images available of the patient *P*, and $N_{rec}$ is the fraction of $N_{P}$ images correctly classified. The Patient Level Accuracy (PLA) is defined as follows:

(2) $$PLA=\frac{\sum Patient\;Score}{Total\;Number\;of\;patients}.$$

Considering the images of the benign class as positive and the images of the malignant class as negative, the Patient Score is defined as the accuracy for a single patient. PLA is an average of the Patient Scores, evaluated as anaverage among the patients.

### 3.2. The Proposed Neural Network Architecture

The general architecture of a deep neural network image classifier can be viewed as a stack comprising a feature extraction part, typically composed of pre-trained convolutional layers, followed by a set of fully connected (FC) layers that implement a Multi-Layer Perceptron (MLP) architecture and serve as a classifier. Among all the layers, only the final layer is trained specifically for network specialisation.

Sometimes in these deep architectures, the last convolutional layer is followed by a “squeeze” operation that precedes the fully connected part of the architecture. The MLP layers have decreasing dimensions from thousands of units to the number of classes. Connecting a layer with dimension *H* to a layer with dimension *D* units in a fully connected architecture generates an H×D weight matrix. As explained in [36], introducing a linear layer *K*, with K<<D and K<<H, results in two matrices H×K and K×D and in a number of weights K×(H+D). The reduced number of weights result in a faster training and can beneficial.

In our architecture, we used a linear layer as a filter for the “signals” generated by the feature extraction lower layers. The training of this linear layer is aimed at separating the input of different classes and can significantly improve the classification results. We perform this training using the metric learning technique implemented with the triplet network learning paradigm; separating the two classes allowed us to remove the MLP layers and use a simpler classifier.

**Figure 2:**
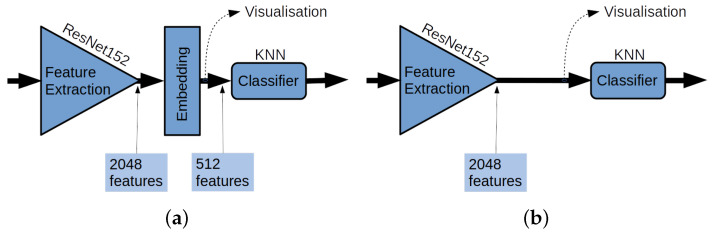
A representation of the classification network proposed in this work: (**a**) The architecture with the embedding layer. (**b**) The architecture without the embedding layer used in the ablation study.

The network proposed in this work uses a ResNet152 network [37], pre-trained on the ImageNet dataset [5] as feature extractor (indicated in Figure 2). These features are linearly projected onto a lower-dimensional space embedding layer (see the Embedding block in Figure 2a). The number of features generated by the ResNet152 is 2048, and the size of the embedding layer is 512. The resulting embedding provides a data representation that is so effective that the MLP layers can be substituted by a simpler *k*-NN classifier, as shown in Figure 2a. In the ablation study (see Figure 2b), we will demonstrate that without the linear embedding, the results are noticeably worse.

The feature extraction of the proposed model is not interpretable or explainable, since ResNet is a very complex model and is already trained via transfer learning.

The training procedure of the linear layer is aimed to transform the feature space, obtained from the feature extraction layer, in a lower dimension space where images of the same class are near each other and images of different classes are taken apart. In this training mechanism, we are trying to confirm underlying information in the training set: images of the same class should have similar characteristics and they should not share features with images of another class.

### 3.3. Metric Learning

Deep metric learning aims to derive effective embeddings from the input data using one or more neural networks and an optimisation strategy based on a chosen distance. In this field, the most-used architectures are the siamese networks [38] and the triplet networks [39]. Both models are constituted by neural networks with shared weights: siamese networks use two neural networks while the triplet network, which we used in this work, has three neural networks.

**Figure 3:**
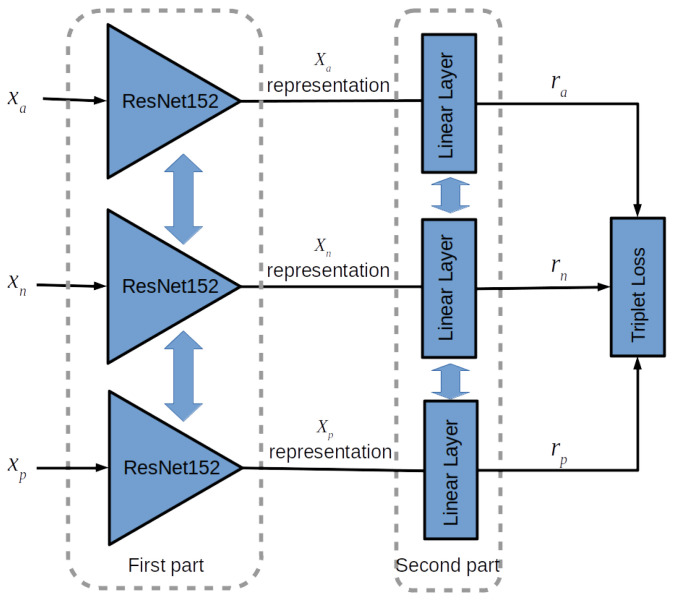
A representation of the network; the vertical double arrow indicates the weights sharing.

Figure 3 reports a representation of the training phase; in this figure three deep networks ResNet152 produce the representations $x_{a},x_{p},x_{n}$ of the three inputs: $x_{a}$ corresponds to the anchor example, $x_{p}$ is the representation of the positive example, an input of the same class of $x_{a}$, and $x_{n}$ is the negative example, an input of a different class. The embedding layer will transform the representations $x_{*}\in {ℜ}^{n}$ to the embeddings $r_{*}\in {ℜ}^{n^{\prime }}$.

Given a distance metric *d*, during training the shared weights of the embedding layer are adjusted so that the value $d(r_{a},r_{n})$ is greater than a prefixed margin *m* w.r.t. the distance $d(r_{a},r_{p})$. The distance *d* and the margin *m* are parameters of the model. The most-used distances are defined using the cosine function or euclidean distance. The representation with three networks and shared weights, like the one in Figure 3, is a common way to indicate the training procedure; however, it is important to point out that the implementation uses a single network receiving the three inputs organised into a sequence, then collecting and storing the values to calculate the global result. The loss function used for training the model is the so-called triplet margin loss [40], defined as:

(3) $$l_{triplet}=\max\left\{0,d\left(r_{a},r_{p}\right)-d\left(r_{a},r_{n}\right)+m\right\}.$$

During the training phase a mining strategy searches—inside the input mini batch—the most effective triplets to update the model. There are different mining strategies [40]; here we adopt the so-called semi-hard mining strategy, defined as:

(4) $$d\left(r_{a},r_{p}\right)<d\left(r_{a},r_{n}\right)<d\left(r_{a},r_{p}\right)+m.$$

This strategy chooses the negative sample to be farther away from the anchor, with respect to the positive, but always bounded by the margin *m*. As a consequence, the network’s loss is bounded by the margin *m*.

### 3.4. Interpretability of the Proposed Model

The interpretability that distinguishes our proposed paradigm belongs to the category of interpretable methodology based on visual analytics [41].

**Figure 4:**
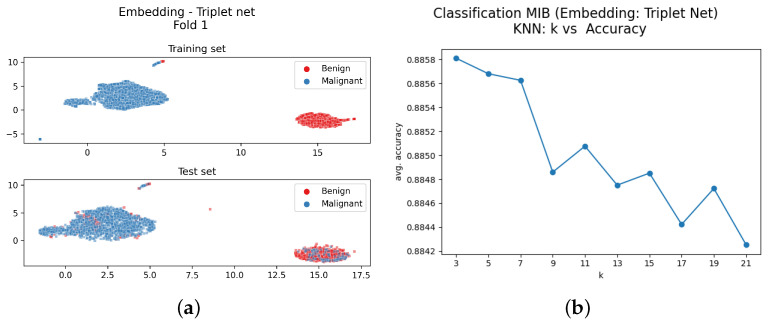
Classification MIB: (**a**) Bi-dimensional representation of the input dataset both training (upper part of Figure 4a) and test set (bottom part of Figure 4b); (**b**) Number of neighbours vs. average accuracy.

The deep metric learning network finds a proper embedding, where the mappings of all the items to classify can be visualised in two or three dimensions using a dimensionality reduction algorithm. One possibility that we have adopted in this paper is the use of Uniform Manifold Approximation and Projection (UMAP) [42], which accomplishes dimensionality reduction using Riemannian geometry and algebraic topology. Metric learning and UMAP introduce a first level of interpretability to the model since they enable the visualisation of the training images that are close to the test ones, and consequently perform neighbour detection (see Figure 4a for an example).

**Figure 5:**
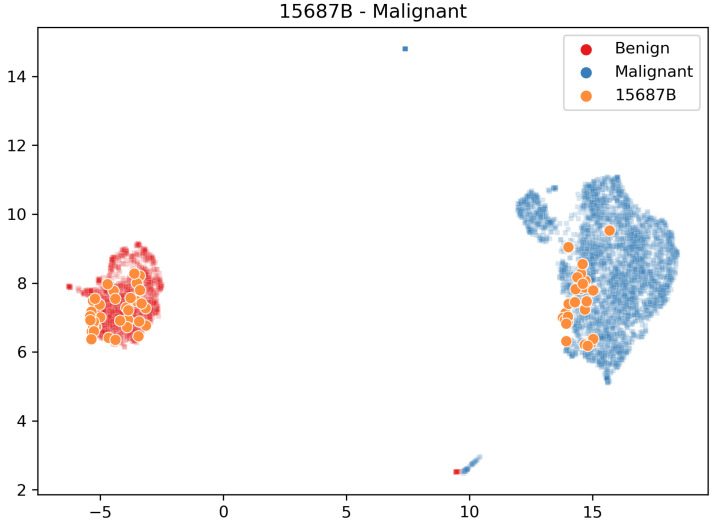
Position of the embedded images of the test patient 15687B in the embedding space w.r.t. the training set embedded images. Our system does not correctly classify most images relating to this patient.

The visualisation of the embedding could also be beneficial to study the distribution of the images related to a specific chosen patient (see Figure 5).

**Figure 6:**
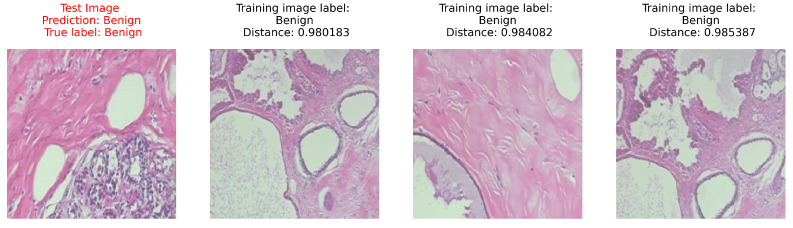
An example of correct classification of a benign image. The test image is on the (**left**); its nearest neighbours with the associated distance are on the (**right**).

The second level of interpretability is introduced by a classifier that clearly maps the input to the output, such as a linear classifier, a Support Vector Machine, or a simple *k*-NN, like the one we adopted here. Unlike the others, with *k*-NN, the nearest neighbors can be used to visually estimate the support for a prediction, providing human-interpretable explanations of predictions. Furthermore, it is always possible for each classified image to display the training images that led to the class labeling (see Figure 6). We find this option very useful as part of a decision support system, where analysts can have interpretable information about the suggested output.

The performances of the proposed model are similar, if not better, to those of other classification systems of the same kind, confirming that the interpretability can be obtained without compromising the performances [7].

### 3.5. Experimental Setup

Experiments have been performed using a workstation equipped with a 12th Gen Intel^®^ Core™ i9-12900KS and an Nvidia 3090 Ti GPU. The total amount of system memory is 32 Gbyte of DDR5. The GPU is also supplied with 24 Gbyte of DDR5 memory and adopts a CUDA parallel computing platform. The operating system is Ubuntu 22.10. We used the TensorFlow Python library [43] to carry out the experiments. The training time for the MIB configuration is about 11 min, while for the MSB configuration about 12 min (3 min for each magnification). We have trained the network for 20 epochs with a mini-batch size of 32 and using the Adam optimisation algorithm [44] with a learning rate of $1\times 10^{-5}$ and weight decay factor of $1\times 10^{-4}$. All the images were resized to 224×224.

## 4. Results

The classification performances obtained with the proposed architecture in both MIB and MSB tasks are reported using the PLA approach. In order to justify the Embedding Layer, an ablation study was carried out, which is described in Section 4.2, using the same classification tasks with the architecture in Figure 2b. Finally, an example of the efficacy of the projection is reported in Section 4.3.

### 4.1. Results with the Proposed Architecture: Triplet Net Embedding

The proposed architecture has been used to classify the BH images in the Magnification Independent (MIB) and Magnification Specific (MSB) fashion. The train/test split used is the 5-fold split proposed by the authors of the dataset.

The training with the MIB procedure uses all the available images in the training set regardless of the magnification factor. After the training of the embedding system, the UMAP procedure was used to visualise the images as points in the embedding space. Then a set of trials was carried out to optimise the k-nearest neighbourhood value. The UMAP visualisation and the plot of the accuracy vs. k-values are in Figure 4; the lower section of Figure 4a shows the embedding of the test images. The upper part of Figure 4a shows that the two clusters of the embedding points obtained from the training images are well-separated. The left cluster collects all the malignant training image projections, and the right cluster collects the benign ones. The same configuration can be observed at the bottom of the sub-figure that reports the test image embeddings. It can be noticed that both sets present two well-separated clusters, and no image is mapped outside these specific areas. Even the misclassified test images are represented as dots in the wrong cluster; there is only a small detached cluster of images on the upper part of the figure.

The plot in Figure 4b shows that the value of *k* has very little influence on the accuracy results: The accuracy change from k=3 to k=21 is less than 1/100.

The PLA accuracy values are in Table 3, compared with the results obtained by Bayramoglu et al. [16] and Sun et al. [17]. The proposed method shows a better performance while requiring a simpler network.

**Figure 7:**
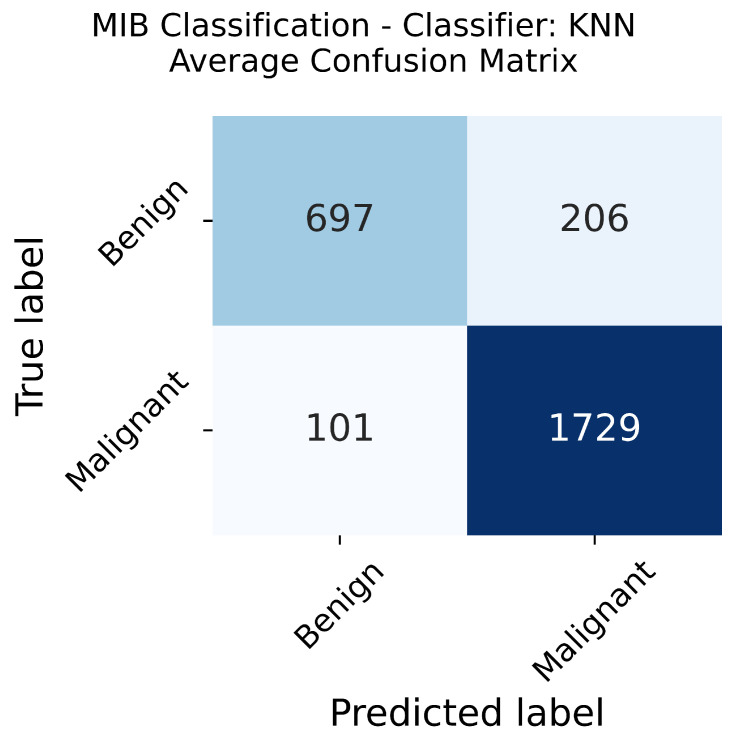
MIB Classification, average confusion matrix.

The confusion matrix is averaged over five folds and is reported in Figure 7. It shows that the number of malignant images misclassified as benign is less than that of benign images misclassified as malignant. This point is crucial in the medical domain since a malignant tumor will receive more attention and probably require more exams than a benign one, and the error has a significant probability of being corrected.

**Figure 8:**
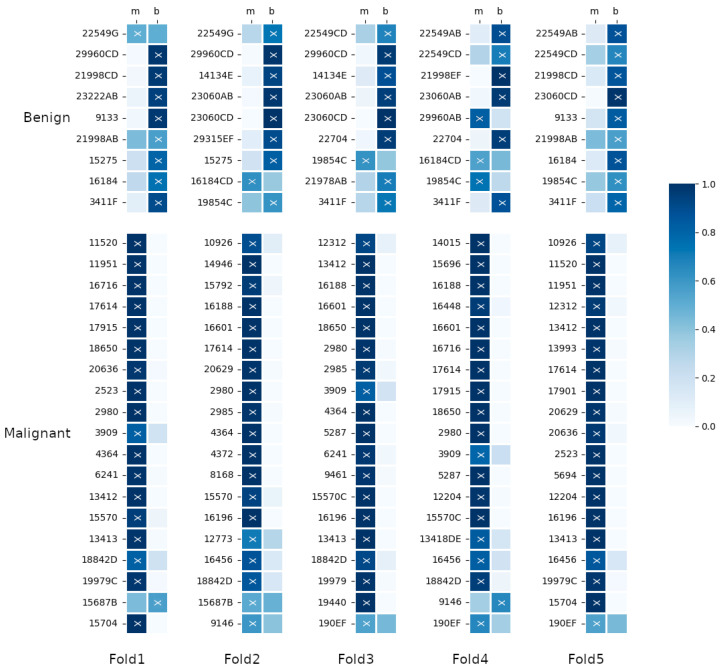
Classification results for each test fold. The colour inside each cell represents the ratio between the image labelled as malignant (benign) and the total number of images of the relative patient. The assigned label is presented as a cross. The ground truth is represented according to the rows.

Figure 8 reports the classification results for each patient of the five folds. Using a blue scale, the ratio between the images labelled as malignant (benign) and the total number of images for that patient is presented. The second level of interpretability is introduced by a classifier that clearly maps the input to the output, such as a linear classifier, a Support Vector Machine, or a simple *k*-NN, like the one we adopted here. Unlike the others, with *k*-NN, the nearest neighbors can be used to visually estimate the support for a prediction, providing human-interpretable explanations of predictions. Furthermore, it is always possible for each classified image to display the training images that led to the class labeling (see Figure 6). We find this option very useful as part of a decision support system, where analysts can have interpretable information about the suggested output.

The assigned class is represented with a cross. The ground truth is presented according to the rows. The first nine rows are from the benign class and the remaining are from the malignant class. In the figure, we can see that 3 patients, 19854C,9146, and 15687B, are classified differently in the five folds. These samples show that the correct or incorrect classification is heavily influenced by the composition of the network’s training set, which requires further investigation. The images of these patients are distributed in both clusters (for example, see Figure 5 for patient 15687B), and the patient classification changes according to the fraction of images correctly classified.

Finally, we notice that each fold has a different error rate: Fold 5 has no errors, while Fold 2 and 3 have one error, Fold 1 has two errors and Fold 4 has 4 errors.

MSB classification is obtained by training four classifiers, one for each magnification factor. Also, in this case, we obtained two well-separated clusters for each training, and the k value has little influence (less than 2/100) on the accuracy values, figures are not reported in this case; we selected k=3 for classification.

The PLA is reported in Table 1, and compared with the other methods cited in Section 2.2.

Note that to evaluate the performance of an image classification system, the widely used metrics are accuracy and F1-Score. According to us, in histopathological image classification, the metric that best simulates reality is patient-based accuracy (PLA); indeed, the pathologist makes the diagnosis by evaluating images of different patients. For completeness, we have reported in Table 4 and Table 5 the values of the other metrics we calculated for the proposed approach: precision, recall, F1-score and the area under the ROC curve (AUC).

### 4.2. Ablation Study: ResNet152 Embedding

The ablation study was conducted to motivate the use of the embedding layer. The new considered model is depicted in Figure 2b. Cancelling the linear layer after the ResNet152 model was used as a feature extractor, embeddings in the metric space were not generated, and the extracted features were used to train the *k*-NN classifier. The visual representation of the images in the 2048-dimensions space is obtained using the UMAP method [42].

**Figure 9:**
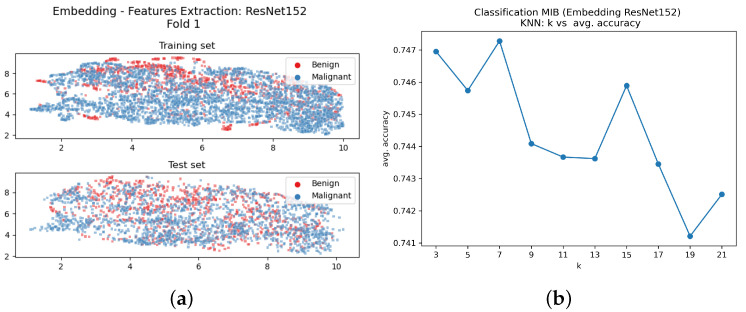
Ablation study with classification MIB: (**a**) Bi-dimensional representation of the input dataset; (**b**) number of neighbours vs. average accuracy.

Figure 9 reports the visualisation of the embeddings in the MIB case, together with the plot of accuracy vs. k value. We can notice that the embedded training or test points are not separated into two clusters, and the k value still has little influence on the accuracy value. The same results can be observed for the MSB classification (results not shown), but in order to obtain the best performance, the k value must be varied for each magnification; the chosen values were 3,7,5 and 9 for the magnification values 40×, 100×, 200× and 400×, respectively. The obtained PLA results are reported in Table 6 for MIB and MSB cases.

### 4.3. Mapping Other Set Images in the Embedded Space

The purpose of the embedding layer is to map the input information into a space that makes simple and effective the classification process and, to support this point, we used a very simple classifier, still obtaining a very good classification accuracy. If we input a new histopathology image, we expect that the system will correctly classify it as malignant or benign, using, at its best, the image features obtained from the lower layers.

The embedding layer was trained with breast cancer histopathology images, and the whole system has knowledge about this kind of images. According to this consideration, the system should not recognise any other kind of image.

Of course, any image can be mapped in the embedding space and the *k*-NN will always provide an output according to the k neighbourhoods. At the same time, we can check the cluster, learned from the training process, where the image is placed.

**Figure 10:**
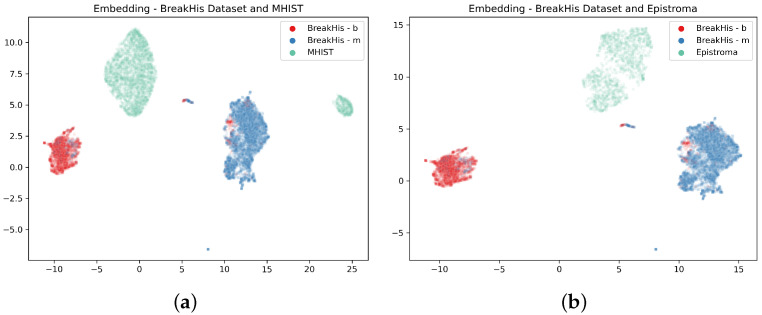
UMAP representation of the embeddings of the BreakHis with other histology image datasets; (**a**) The embedding of the MHIST dataset obtained using the triplet net trained on the BreakHis dataset; (**b**) The embedding of the Epistroma dataset obtained using the triplet net trained on the BreakHis dataset.

If we input a random image into the system, it would be desirable that the system answer that the features were not appropriate to classify it meaningfully. We expect that a random image should be in an area of the embedding space far from the embedding of the BH images. In order to test this hypothesis, we visualise the position where the system, trained with BreakHis dataset, maps the images of two different histological image datasets: The MHIST dataset [45] (Minimalist HISTopathology contains images of colon-rectal polyps) and the Epistroma dataset [46,47] (that contains histopathology images belonging to two tissue types: epithelium and stroma). These image sets were selected because they are of the same kind (histology images) but from a different origin. Figure 10a,b show the results: The embedding point clouds are far away from the BH image projection.

## 5. Discussion

There is a common idea that interpretability should be paid with a substantial loss of performance. This claim can be found in [48]; however, C. Rudin argued in [7] that there is not evidence supporting this statement. In the last section, we show that this is not necessarily true; it is possible to support interpretability without a significant loss of performance. In the following, we will discuss the result comparison with the state of the art and the interpretability of the proposed framework.

### 5.1. The Performances of the System

There are many works on the classification of BH dataset, those reported in Section 2 are only the one that contains enough information for a comparison using the PLA metrics, the only metrics that make sense in this kind of studies. According to these premises, the presented work can be compared with very few works on the MIB classification, while there are more works for the MSB classification. Our results for MIB are better than the state-of-the-art results, as reported in Table 3, and we notice that the accuracy values in the MSB task are somewhat lower than the ones in the state-of-the-art works. In Table 1, we report the results from seven different works; the comparison shows that only in 40× magnification our results are less than 5% below the work of Sudharshan et al. [26]; for other magnification values, the accuracy values are just 2 or 3% below. In the MSB classification, it is necessary to train one neural network for each magnification value, and separating the training images into the different magnification sub-classes results in a smaller number of training images for each neural network. However, in our experiments, this effect is not recovered using augmentation, and this requires more investigations. All the methods that perform better than our proposal involve complex learning paradigms such as classifier combination (for the case of Spanhol et al. [20] or Multiple Instance Learning (Sudharshan et al. [26]) or some kind of specific pre-processing (re-scaling for the case of Song et al. [23]). While benefits can be observed from the performances, they remain not very interpretable.

The proposed method is based on a clear and simple mechanism, the ablation study shows that the cluster separation is only due to the presence of the linear layer and the metric learning training, and this is clear comparing Figure 4a and Figure 9a. Table 6 also shows a significant performance drop both in MIB and MSB classification.

### 5.2. The Interpretability Aspects

The interpretability of the proposed method is based on the set of information that can be obtained at the end of the embedding layer and from the *k*-NN classification module. The metric learning layer rearranges the embedding space while reducing the number of dimensions. In the new embedding space, the training images are meaningfully clustered, and the visualisation allows the user to understand the system response. This visualisation constitutes the first set of information that can help the user to understand the first processing steps. Test images are clustered in the same areas of the training ones; this happens for both classification methods MIB and MSB. The visualization of the training image in the embedding space in Figure 4a shows that metric learning creates a sort of “areas” where input images should be projected. The visualisation of the embedding space is also useful during the classification of a new input. Observing the position of the images of a new patient in the embedding space, such as the ones reported in Figure 5, the user can check if this input is near the embeddings of the training images set, meaning that the new input came from the same distribution of the training images.

Finally, the same visualisation can spot when the network is not able to process an input image. Figure 10 shows what happens if the input is an out-of-distribution image set. In this case, the images are embedded in points outside the two clusters obtained from the BH training images. In cases like this, the *k*-NN classifier will assign a class to the input based on the nearest images, but the visualisation shows that the features of the inputs are different from the ones of the training images. This consideration allows an examiner to say that the system classification output will have no meaning since the images are outside what the system “knows”.

Focusing on the classification algorithm, notice that *k*-NN is considered one of the naturally interpretable classification algorithm. For each classification result is always possible to visualise the train images that produce the result and, thanks to the application of metric learning, the number of neighbourhoods is very low, less than the one obtained in the ablation study.

This organisation of the embedding space allows using a *k*-NN classifier with a very low k, so that an user can visualise the neighbourhood of a test image and easily spot the similarity and the differences with its neighbourhood. An example of classification in MIB is reported in Figure 6, with the neighbour images. This figure shows the three nearest neighbourhood images used to label the test image (left image). As pointed out in the book [49], the interpretation of the classification results is translated to the interpretation of the neighbourhood images, and a domain expert can give the right meaning to the images similarity.

## 6. Conclusions

The high performances obtained with the DNN in the medical domain are confirmed by many studies and applications, and histopathology image classification is one of these applications. However, there are two main problems connected with many of these studies: The first is connected with the train and test set; not all the works consider that the focus is on the patient, not on the images, and the patient should be completely unknown for the system undergoing testing. The second problem is related to the interpretability of the supporting systems; in the medical domain the answer of a supporting system should always be followed with an explanation of the decisions steps.

In this paper, we have presented a classification model based on a deep neural network and an interpretable classifier, which take into account the above-mentioned considerations. In particular, we used a triplet network based on a ResNet152 as a deep network, which allows us to map the input samples into an embedding space learned to represent images of different classes as separate clusters. The representation in this embedding space is viable for the comparison of a test image with its neighbourhood, providing an explanation of the classification as compared with images of the same kind. The final classification is performed with a simple classifier (*k*-NN) since the input information is represented through the neural network. Interestingly, only a few layers of the model are trained, while the majority of the network is pre-trained and does not need to be updated.

The results obtained with this technique are better than those obtained with the ad-hoc full-trained deep neural networks and allow the user to visualise the representation of the input image together with the most similar training image. The proposed technique can be used in many classifier architectures based on representation/classification schema. Future research will be focused on the analysis of the neighbourhood images in order to find the details that can be considered characteristic of a specific class.

## Figures and Tables

**Table 1:** Triplet net embedding: MSB classification (PLA) results and comparison with the other methods.

Method	40×	100×	200×	400×
Sun et al. [17]	87.51±4.07	89.12±2.86	90.82±3.31	87.10±3.80
Spanhol et al. [19]	90.0±6.7	88.4±4.8	84.6±4.2	86.1±6.2
Spanhol et al. [20]	84.0±6.9	83.9±5.9	86.3±3.5	82.1±2.4
Benhammou et al. [21]	87.6±3.9	82.4±2.7	86.1±0.7	79.7±3.2
Song et al. [23]	90.0±3.2	88.9±5.0	86.9±5.2	86.3±7.0
Song et al. [25]	90.2±3.2	91.2±4.4	87.8±5.3	87.4±7.2
Sudharshan et al. [26]	92.1±5.9	89.1±5.2	87.2±4.3	87.8±5.6
Proposed method (*k*-NN)	87.60±3.92	88.17±3.86	89.37±3.26	85.98±2.78

**Table 2:** The BreakHis dataset: for each patient the set of images is divided in magnifications 40×, 100×, 200×, 400×.

Benign	Cancer Types	No. of Images	No. of Patients
	Adenosis	444	4
	Fibroadenoma	1014	10
	Tubular Adenoma	453	7
	Phyllodes Tumour	569	3
Total		2480	24
**Malignant**	**Cancer Types**	**No. of Images**	**No. of Patients**
	Ductal Carcinoma	3451	38
	Lobular Carcinoma	626	5
	Mucinous Carcinoma	792	9
	Papillary Carcinoma	560	6
Total		5429	58

**Table 3:** Triplet net embedding: MIB classification results and comparison with the other methods.

Method	PLA
Bayramoglu et al. [16]	82.13
Sun et al. [17]	88.40±4.10
Proposed method (*k*-NN)	88.90±2.41

**Table 4:** Triplet net embedding: MIB classification metrics.

Accuracy	Precision	Recall	F1-Score	AUC
88.71±2.10	88.24±2.78	85.87±2.02	86.84±2.28	85.87±2.02

**Table 5:** Triplet net embedding: MSB classification metrics.

Metric	40 ×	100 ×	200 ×	400 ×
Accuracy	84.60±3.53	86.34±3.13	88.70±3.22	84.98±3.17
Precision	85.48±4.45	87.16±3.40	88.36±3.20	84.86±2.78
Recall	94.42±2.36	95.24±3.13	94.79±4.42	94.04±4.50
F1-Score	89.15±2.51	90.31±2.25	91.90±2.34	89.23±2.36
AUC	82.36±3.67	83.14±3.70	87.68±4.12	81.67±2.94

**Table 6:** Ablation study: Magnification Independent (MIB) and Magnification Specific (MSB) classification with *k*-NN.

		PLA-*k*-NN
MIB		75%
MSB	40×	79%
	100×	78%
	200×	86%
	400×	80%

## Data Availability

The BreakHis dataset is publicly available at https://web.inf.ufpr.br/vri/databases/breast-cancer-histopathological-database-breakhis/ (accessed on 1 June 2022). The MHIST dataset is available at https://bmirds.github.io/MHIST/ (accessed on 1 June 2022). The Epistroma dataset is publicly available at http://fimm.webmicroscope.net/Research/Supplements/epistroma (accessed on 1 June 2022). The source code is available at https://github.com/Calder10/BreakHis-MIB-MSB-Classification-Metric-Learning (accessed on 1 June 2022).

## Abbreviations

The following abbreviations are used in this manuscript:

BH	BreakHis (dataset)
CNN	Convolution Neural Network
CVF	ConvNet-based Fisher Vectors
DNN	Deep Neural Network
FC	Fully Connected
ILA	Image Level Accuracy
*k*-NN	k Nearest Neighbour
MI	Magnification Independent
MIB	Magnification Independent Binary (classification)
MLP	Multi Layer Perceptron
MS	Magnification Specific
MSB	Magnification Specific Binary (classification)
PFTAS	Parametric-Free Threshold Adjacency Statistics
PLA	Patient Level Accuracy
AUC	Area under the Roc Curve
UMAP	Uniform Manifold Approximation and Projection
